# Effects of Eltrombopag on In Vitro Macrophage Polarization in Pediatric Immune Thrombocytopenia

**DOI:** 10.3390/ijms22010097

**Published:** 2020-12-24

**Authors:** Alessandra Di Paola, Giuseppe Palumbo, Pietro Merli, Maura Argenziano, Chiara Tortora, Luisa Strocchio, Domenico Roberti, Claudia Santoro, Silverio Perrotta, Francesca Rossi

**Affiliations:** 1 Department of Experimental Medicine, University of Campania “Luigi Vanvitelli”, 80138 Naples, Italy; alessandra.dipaola92@gmail.com (A.D.P.); maurargenziano@gmail.com (M.A.); 2 Department of Haematology, Bambino Gesù Hospital, 00165 Rome, Italy; giuseppe.palumbo@opbg.net (G.P.); pietro.merli@opbg.net (P.M.); luisa.strocchio@opbg.net (L.S.); 3 Department of Woman, Child and General and Specialist Surgery, University of Campania “Luigi Vanvitelli”, 80138 Naples, Italy; chiara.tortora@unicampania.it (C.T.); domenico.roberti@unicampania.it (D.R.); silverio.perrotta@unicampania.it (S.P.); 4 Department of Mental and Physical Health and Preventive Medicine, University of Campania “Luigi Vanvitelli”, 80138 Naples, Italy; claudia.santoro@unicampania.it

**Keywords:** immune thrombocytopenia, eltrombopag, macrophages, M1, M2, inflammation, macrophage polarization

## Abstract

Immune Thrombocytopenia (ITP) is an autoimmune disease characterized by autoantibodies-mediated platelet destruction, a prevalence of M1 pro-inflammatory macrophage phenotype and an elevated T helper 1 and T helper 2 lymphocytes (Th1/Th2) ratio, resulting in impairment of inflammatory profile and immune response. Macrophages are immune cells, present as pro-inflammatory classically activated macrophages (M1) or as anti-inflammatory alternatively activated macrophages (M2). They have a key role in ITP, acting both as effector cells, phagocytizing platelets, and, as antigen presenting cells, stimulating auto-antibodies against platelets production. Eltrombopag (ELT) is a thrombopoietin receptor agonist licensed for chronic ITP to stimulate platelet production. Moreover, it improves T and B regulatory cells functions, suppresses T-cells activity, and inhibits monocytes activation. We analyzed the effect of ELT on macrophage phenotype polarization, proposing a new possible mechanism of action. We suggest it as a mediator of macrophage phenotype switch from the M1 pro-inflammatory type to the M2 anti-inflammatory one in paediatric patients with ITP, in order to reduce inflammatory state and restore the immune system function. Our results provide new insights into the therapy and the management of ITP, suggesting ELT also as immune-modulating drug.

## 1. Introduction

Immune Thrombocytopenia (ITP) is an autoimmune disease characterized by severe thrombocytopenia, due to autoantibodies-mediated platelet destruction [1]. An important impairment of the immune system is observed in ITP patients [2,3,4]. In particular, macrophages play a key role acting both as effector cells, phagocytizing platelets, and as antigen presenting cells, stimulating auto-antibodies against platelets production by B cells [5,6,7].

Macrophages are phagocytic mononuclear cells involved in inflammatory processes and in immune response. They can be present in two different activation phenotypes: the classically activated macrophages (M1) and the alternatively activated macrophages (M2) [8,9,10]. M1 macrophages are activated by Tumor Necrosis Factor-α (TNF-α), Interferon-γ (IFN-γ), and bacterial lipopolysaccharide (LPS). They exert pro-inflammatory, anti-tumor, and anti-microbial functions, releasing high levels of pro-inflammatory cytokines, such as TNF-α, Interleukin-6 (IL-6), Interleukin-1β (IL-1β), and Nitric Oxide Synthase (iNOS) [8,9,10,11]. M2 macrophages are activated by Interleukin-4 (IL-4), Interleukin-13 (IL-13), Interleukin-10 (IL-10), and by Phosphatidylinositol 3-kinase-AKT-mammalian target of rapamycin (PI3K-Akt-mTOR) signaling pathway. They exert anti-inflammatory and immunosuppressive activities, by releasing anti-inflammatory cytokines, such as IL-10 and Transforming Growth Factor-β (TGF-β), and have also pro-angiogenic and pro-fibrotic properties, promoting tumor progression [8,9,10,11]. M2 alternatively activated macrophages also express Cluster of Differentiation 206 (CD206). It is a mannose receptor which provides the clearance of glycoproteins involved in allergy or inflammation, and it is responsible for production of pro and anti-inflammatory cytokines. In ITP, an imbalance of M1/M2 ratio has been observed, with a prevalence of M1 macrophages, responsible for an increased release of pro-inflammatory cytokines. This alteration together with an elevated ratio between T helper 1 and T helper 2 lymphocytes (Th1/Th2) determines a deficient immune response [7,8,12,13]. Th1 are responsible for macrophage activation, platelet phagocytosis, and pro-inflammatory cytokines production [5,14]. Corticosteroids represent the first-line therapy in ITP, but their prolonged use could cause important side effects, such as weight gain, hypertension, and diabetes [4,15,16,17,18].

Eltrombopag (ELT) is an orally available thrombopoietin receptor agonist (TPO-RA), licensed for chronic ITP treatment in both pediatric and adult patients when the first-line therapy or splenectomy fail [19,20,21,22]. It principally promotes platelets production by stimulating hematopoietic stem cells [20] and it also shows immunomodulating properties. Indeed ELT improves T and B regulatory cells activity [23,24], suppressing T-cell responses to platelet auto-antigens [23,25]. Moreover, Liu et al. demonstrated that ELT is also able to inhibit monocyte activation by reversing Fcγ receptors toward an inhibitory phenotype. [26]. However, the underlying mechanism is still under study. ELT also has iron chelating properties, binding iron (III), the main intracellular form [27,28]. The M1 pro-inflammatory macrophages are characterized by an increase in iron internalization and consequently of its intracellular concentration (Fe^3+^) [29,30]. Iron accumulation induces the expression of pro-inflammatory cytokines and the production of reactive oxygen species (ROS), contributing to the inflammatory state [30]. M2 phenotype is instead responsible of iron release. The low iron intracellular concentration induces the inhibition of pro-inflammatory cytokines expression and a reduction of iNOS expression [30].

Considering the impairment of M1/M2 ratio in ITP with a prevalence of the M1 pro-inflammatory phenotype, in this study, we proposed a new possible mechanism of action of ELT in regulating macrophage phenotype polarization. In particular, we evaluated ELT capability to induce macrophage phenotype switch from the M1 pro-inflammatory phenotype to the M2 anti-inflammatory type in ITP pediatric patients, in order to reduce inflammatory state and restore the immune system function.

## 2. Results

### 2.1. Effect of Eltrombopag on iNOS and CD206 Proteins Expression

**Figure 1:**
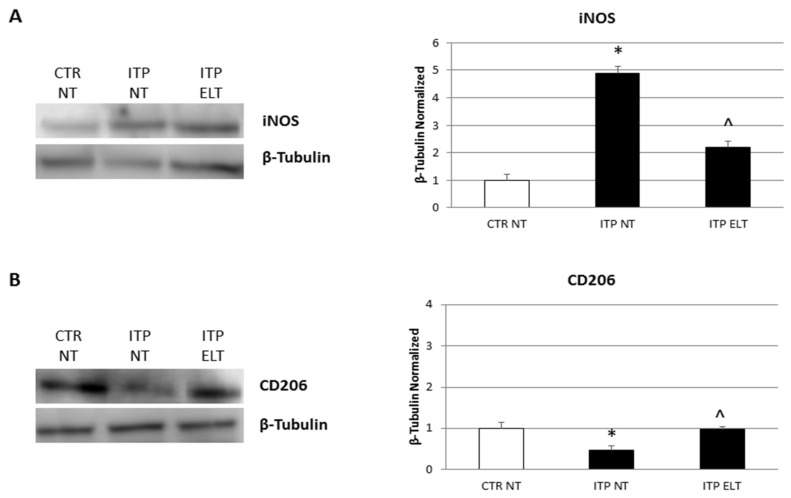
Effect of Eltrombopag (ELT) on Nitric Oxide Synthase (iNOS) and Cluster of Differentiation 206 (CD206) protein expression. (**A**) iNOS protein expression levels in Immune Thrombocytopenia (ITP) macrophages compared with CTR (control) macrophages, determined by Western Blot, starting from 20 μg of total lysates after treatment with Eltrombopag (6 μM). (**B**) CD206 protein expression levels in ITP macrophages compared with CTR macrophages, determined by Western Blot, starting from 20 μg of total lysates after treatment with ELT (6 μM). The most representative images are displayed. The proteins were detected using Image Studio Digits software (LI_COR Biotechnology ®, Lincoln, Nebraska USA)and the intensity of immunoblots compared to the untreated control, taken as 1 (arbitrary unit), were quantified after normalizing with respective loading controls for the housekeeping protein β-Tubulin. Histogram shows iNOS (**A**) and CD206 (**B**) expression levels as the mean ± S.D. of independent experiments on each individual sample. A *t*-test was used for statistical analysis. * indicates *p* ≤ 0.05 compared to Non-treated Control (CTR NT), ^ indicates *p* ≤ 0.05 compared to ITP NT.

To evaluate protein expression levels of the M1 polarization marker, iNOS, and of the M2 polarization marker, CD206, we performed Western Blot. Firstly, we compared iNOS and CD206 expression levels in macrophages obtained from ITP patients (ITP NT) with macrophages obtained from healthy donors (CTR NT). Biochemical analysis revealed that, in ITP NT, the levels of iNOS and CD206 are, respectively, higher and lower compared with CTR NT in a statistically significant manner, indicating that, in ITP patients, there was a prevalence of M1 macrophage phenotype. Then, we evaluated the effect of ELT (6 µM) treatment on both macrophage polarization markers, and we observed a decrease of iNOS and an increase of CD206 expression levels, both statistically significant, in the treated samples (ITP ELT) compared to the non-treated ones (ITP NT) (Figure 1A,B). These results let us suppose that ELT is able to determine a phenotype switch towards the anti-inflammatory M2 macrophage type.

### 2.2. Effect of Eltrombopag on Pro-Inflammatory Cytokines Release

**Figure 2:**
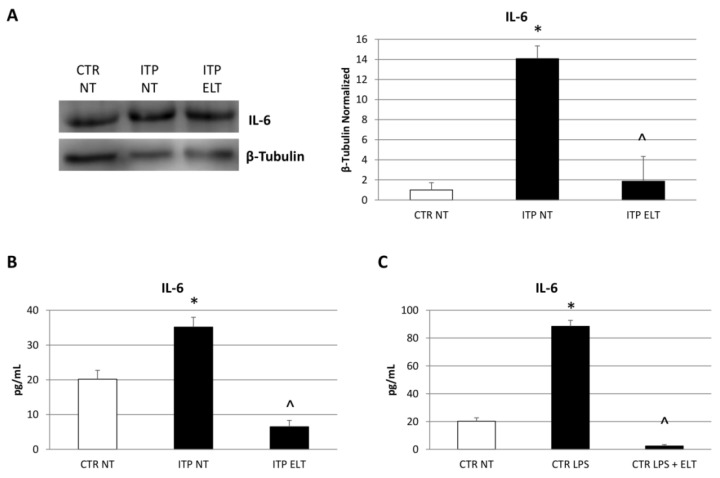
Effect of ELT on Interleukin-6 (IL-6) protein expression and release. (**A**) IL-6 protein expression levels in ITP macrophages compared with CTR macrophages, determined by Western Blot, starting from 20 μg of total lysates after treatment with ELT (6 μM). The most representative images are displayed. The proteins were detected using Image Studio Digits software, and the intensity of immunoblots compared to the untreated control, taken as 1 (arbitrary unit), were quantified after normalizing with respective loading controls for the housekeeping protein β-Tubulin. Histogram shows IL-6 expression levels as the mean ± S.D. of independent experiments on each individual sample. A *t*-test was used for statistical analysis. * indicates *p* ≤ 0.05 compared to CTR NT, ^ indicates *p* ≤ 0.05 compared ITP NT. (**B**) IL-6 concentrations (pg/mL) in ITP macrophages compared with CTR macrophages, determined by enzyme-linked immunosorbent assay (ELISA), after treatment with ELT (6 μM). Histogram shows IL-6 concentration as the mean ± S.D of independent experiments on each individual sample. The cytokines concentration was determined on a standard concentration curve according to the manufacturer’s instructions. A *t*-test wasused for statistical analysis. * indicates *p* ≤ 0.05 compared to CTR NT, ^ indicates *p* ≤ 0.05 compared ITP NT. (**C**) IL-6 concentrations (pg/mL) in ITP macrophages compared with CTR macrophages, determined by ELISA Assay, after treatment with lipopolysaccharide (LPS) (500 nM) and LPS combined with ELT (6 μM). Histogram shows IL-6 concentration as the mean ± S.D. of independent experiments on each individual sample. The cytokines concentration was determined on a standard concentration curve according to the manufacturer’s instructions. A *t*-test was used for statistical analysis. * indicates *p* ≤ 0.05 compared to CTR NT, ^ indicates *p* ≤ 0.05 compared to CTR LPS.

**Figure 3:**
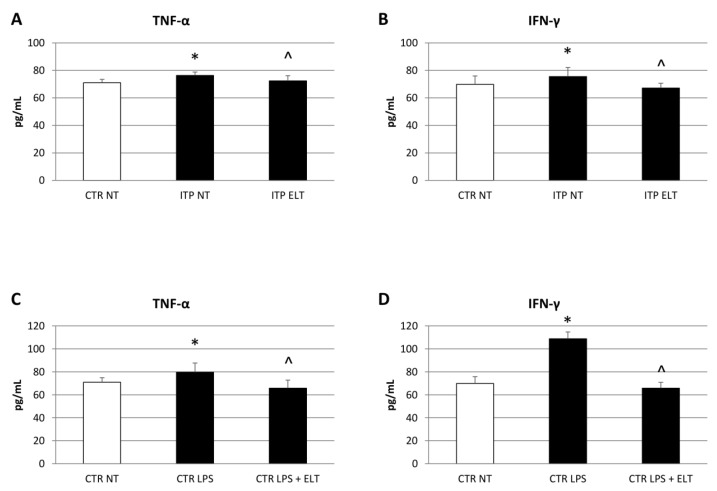
Effect of ELT on Tumor Necrosis Factor-α (TNF-α) and Interferon-γ (IFN-γ) release. (**A**,**B**) TNF-α and IFN-γ concentrations (pg/mL) in ITP macrophages compared with CTR NT macrophages, determined by enzyme-linked immunosorbent assay (ELISA Assay), after treatment with ELT (6 μM). Histogram shows TNF-α and IFN-γ concentrations as the mean ± S.D. of independent experiments on each individual sample. The cytokines concentration was determined on a standard concentration curve according to the manufacturer’s instructions. A *t*-test was used for statistical analysis. * indicates *p* ≤ 0.05 compared to CTR NT, ^ indicates *p* ≤ 0.05 compared ITP NT. (**C**,**D**) TNF-α and IFN-γ concentrations (pg/mL) in ITP macrophages compared with CTR macrophages, determined by ELISA Assay, after treatment with LPS (500 nM) and LPS combined with ELT (6 μM). Histogram shows TNF-α and IFN-γ concentrations as the mean ± S.D. of independent experiments on each individual sample. The cytokines concentration was determined on a standard concentration curve according to the manufacturer’s instructions. A *t*-test was used for statistical analysis. * indicates *p* ≤ 0.05 compared to CTR NT, ^ indicates *p* ≤ 0.05 compared to CTR LPS.

To investigate the effect of ELT (6 µM) on pro-inflammatory cytokines profile, we performed a Western Blot and several enzyme-linked immunosorbent assays (ELISA). Firstly, we analyzed the pro-inflammatory cytokine IL-6 protein expression levels by Western Blot, and we observed a very strong increase of its expression levels in ITP NT compared to CTR NT. Accordingly, ELISA also revealed a statistically significant increase of IL-6 release in ITP macrophages. The administration of ELT (6 µM) induced a significant reduction of IL-6 expression levels and release (Figure 2A,B). We confirmed these data after also stimulating CTR macrophages with LPS (500 ng/mL), indeed after the inflammatory the IL-6 levels were higher after the inflammatory stimulus with LPS (500 ng/mL). ELT (6 µM) was able to restore the inflammatory state, reducing IL-6 release (Figure 2C). Then, we evaluated the release of the pro-inflammatory cytokines TNF-α and IFN-γ. In ITP NT, we observed an increase of their release and that ELT administration induced a statistically significant reduction (Figure 3A,B). In addition, in CTR NT, after LPS (500 ng/mL) stimulation, we revealed an increase of TNF-α and IFN-γ release. The treatment with ELT (6 µM) induced a remarkable decrease of their release, ameliorating the inflammatory state (Figure 3C,D).

### 2.3. Effect of Eltrombopag on Anti-Inflammatory Cytokines Release

**Figure 4:**
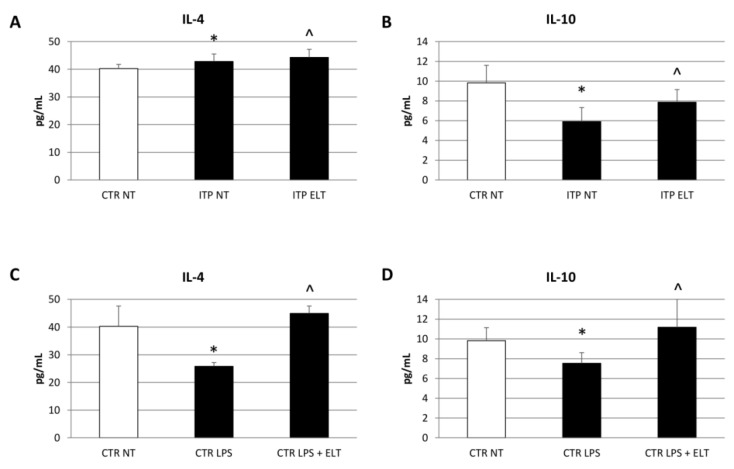
Effect of ELT on IL-4 and IL-10 release. (**A**,**B**) IL-4 and IL-10 concentrations (pg/mL) in ITP macrophages compared with CTR NT macrophages, determined by Enzyme-linked Immunosorbent assay (ELISA Assay), after treatment with ELT (6 μM). Histogram shows IL-4 (**A**) and IL-10 (**B**) concentrations as the mean ± S.D. of independent experiments on each individual sample. The cytokines concentration was determined on a standard concentration curve according to the manufacturer’s instructions. A *t*-test wasused for statistical analysis. * indicates *p* ≤ 0.05 compared to CTR NT, ^ indicates *p* ≤ 0.05 compared ITP NT. (**C**,**D**) IL-4 and IL-10 concentrations (pg/mL) in ITP macrophages compared with CTR macrophages, determined by ELISA Assay, after treatment with LPS (500 nM) and LPS combined with ELT (6 μM). Histogram shows IL-4 (**C**) and IL-10 (**D**) concentrations as the mean ± S.D. of independent experiments on each individual sample. The cytokines concentration was determined on a standard concentration curve according to the manufacturer’s instructions. A *t*-test was used for statistical analysis. * indicates *p* ≤ 0.05 compared to CTR NT, ^ indicates *p* ≤ 0.05 compared to CTR LPS.

We also investigated the anti-inflammatory cytokines IL-4 and IL-10 release by ELISA assays. We observed a reduction of their release in ITP macrophages with respect to CTR macrophages. The administration of ELT (6 µM) induced a statistically significant increase of both IL-4 and IL-10 in supernatant of ITP macrophages, letting us suppose it could ameliorate the inflammatory state (Figure 4A,B). These data were also confirmed in CTR macrophages treated with LPS (500 nM). We observed a reduction of IL-4 and IL-10 concentration in LPS-treated Macro-CTR; after ELT (6 µM) treatment, we revealed an increase of the anti-inflammatory cytokines concentration (Figure 4C,D).

### 2.4. Effect of Eltrombopag on Macrophage Iron Metabolism

**Figure 5:**
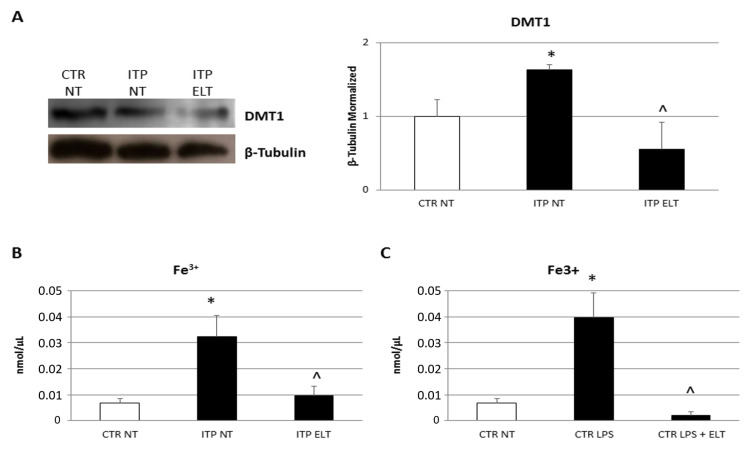
Effect of ELT on divalent metal transporter 1 (DMT1) protein expression and on Iron release. (**A**) DMT1 protein expression levels in ITP macrophages compared with CTR macrophages, determined by Western Blot, starting from 20 μg of total lysates after treatment with ELT (6 μM). The most representative images are displayed. The proteins were detected using Image Studio Digits software, and the intensity of immunoblots compared to the untreated control, taken as 1 (arbitrary unit), were quantified after normalizing with respective loading controls for the housekeeping protein β-Tubulin. Histogram shows DMT1 expression levels as the mean ± S.D. of independent experiments on each individual sample. A *t*-test was used for statistical analysis. * indicates *p* ≤ 0.05 compared to CTR NT, ^ indicates *p* ≤ 0.05 compared ITP NT. (**B**) Fe^3+^ intracellular concentrations (nmol/µL) in ITP macrophages compared with CTR macrophages, determined by Iron Assay, after treatment with ELT (6 μM). Histogram shows Fe^3+^ concentration as the mean ± S.D. of independent experiments on each individual sample. A *t*-test was used for statistical analysis. *indicates *p* ≤ 0.05 compared to CTR NT, ^ indicates *p* ≤ 0.05 compared to ITP NT. (**C**) Fe^3+^ intracellular concentrations (nmol/µL) in ITP macrophages compared with CTR macrophages, determined by Iron Assay, after treatment with LPS (500 nM) and LPS combined with ELT (6 μM). Histogram shows Fe^3+^ concentration as the mean ± S.D. of independent experiments on each individual sample. A *t*-test was used for statistical analysis. * indicates *p* ≤ 0.05 compared to CTR NT, ^ indicates *p* ≤ 0.05 compared to CTR LPS.

In order to understand the effect of ELT on iron metabolism, we performed a Western Blot to evaluate the protein expression level of the iron transporter divalent metal transporter 1 (DMT1). We revealed that, in ITP macrophages, DMT1 protein expression level is higher than in CTR macrophages. ELT (6 μM) induced a reduction of its expression, thus reducing its internalization (Figure 5A). To confirm this data and the iron-chelating property of ELT, we measured the intracellular ferric iron ion concentration (Fe^3+^) by Iron Assay. We observed a strong increase of (Fe^3+^) in ITP NT compared to CTR NT. ELT (6 μM) induced a statistically significant reduction of (Fe^3+^) (Figure 5B). A similar trend was observed in CTR macrophages treated with LPS (500 nM), in which we observed an increase of iron concentration; ELT (6 μM) administration reduced (Fe^3+^) intracellular levels (Figure 5C).

## 3. Discussion

Immune Thrombocytopenia (ITP) is an autoimmune disorder caused by an immune-mediated platelets destruction and characterized by an impairment of the immune system [1,2,3], with a prevalence of M1 macrophage phenotype [8] and an elevated Th1/Th2 ratio [31]. Th1 high levels are responsible for stimulation of macrophages [31,32]. Macrophages are immune cells with a key role in inflammatory processes [8,9,10], and they are present in two distinct states of activation: the classically activated macrophages (M1) and the alternatively activated macrophages (M2) [9,10,11]. While M1 exert pro-inflammatory, anti-tumor, and anti-microbial properties, releasing pro-inflammatory cytokines [8,9,10,11], M2 show anti-inflammatory and immunosuppressive functions, releasing anti-inflammatory cytokines and promoting tumor progression [8,9,10,11]. The prevalence of M1 in ITP is responsible for both disease pathogenesis and impairment in inflammatory and immune responses [8,9,10]. We analyzed the possibility to induce a macrophage phenotype switch towards M2 type, using Eltrombopag (ELT).

ELT is an orally available thrombopoietin receptor agonist already used in chronic ITP patients [19,20,21,22] to stimulate platelet production. It also modulates immune system, reducing Th1 function [9,23,24,25] and inhibiting macrophages activation [26].

According to literature, our results highlighted higher levels of iNOS and lower levels of CD206 in ITP patients macrophages compared with macrophages from heathy donors. iNOS and CD206 are the most used markers to distinguish M1 and M2 phenotypes, respectively [33]. After treating ITP macrophages with ELT, we observed a strong reduction of iNOS expression and a significant increase of CD206 levels, letting hypothesize ELT involvement in macrophages phenotype switch toward the anti-inflammatory and immune suppressive M2 type. The protective role of ELT is supported also by the analysis of cytokine profile before and after its administration. In accordance with other authors [5,8,9,10,32], we observed an impairment in ITP patient cytokine profile, with a prevalence of pro-inflammatory cytokines. In particular, we detected an increase of Interleukin-6 (IL-6), a pro-inflammatory cytokine released by M1 macrophages [8,9,10]. This data confirms the prevalence of M1 phenotype in ITP. ELT administration induced a significant reduction in IL-6 production, very likely related to macrophage polarization from the M1 pro-inflammatory phenotype to the M2 anti-inflammatory one. We also observed an increased production of Tumor Necrosis Factor-α (TNF-α) and Interferon-γ (IFN-γ) in ITP macrophages, confirming once again the impaired cytokine profile in ITP [5,32]. TNF-α and IFN-γ are both pro-inflammatory cytokines; in particular, IFN-γ induces activation of M1 macrophages [9]. Interestingly, ELT treatment determined a reduction of these pro-inflammatory cytokines release, evidently mediating the polarization towards the M2 anti-inflammatory phenotype. To strengthen these results, we analyzed the effect of ELT administration on anti-inflammatory cytokines, Interleukin-4 (IL-4) and Interleukin-10 (IL-10). IL-4 is responsible for M2 macrophages polarization, while IL-10 is produced by M2 phenotype to counteract inflammation [9]. In ITP patients, as expected, their levels are lower than in healthy donors, but, after ELT administration, we observed an increase in their release. Once again, the capability of ELT to induce the M2 profile in ITP macrophages is very evident.

Iron chelation is another newly emerged property of ELT [28]. It is well known that M1 and M2 macrophages are involved in regulation of iron homeostasis with opposite roles. M1 phenotype is characterized by an increase of iron internalization which induces pro-inflammatory cytokines and ROS production [30]. Conversely, M2 macrophages are responsible for iron release and the consequent low iron intracellular concentration induces a reduction of iNOS and pro-inflammatory cytokines expression [30]. We observed an increase of DMT1 protein expression level and of iron intracellular concentration in ITP macrophages. DMT1 is an iron transporter involved in macrophage iron recycling and responsible for iron intake into the cytosol [11,34]. It has been observed that macrophages deficient in functional DMT1 are unable to efficiently recycle iron [34]. ELT administration not only induces iron chelation by reducing its intracellular concentration, but it also significantly reduces DMT1 expression, inhibiting iron intake in the cells. Therefore, these results confirm the well-known iron chelating property of ELT and also suggest, once again, that ELT induces M2 macrophage polarization, inducing a reduction of intracellular iron concentration and of DMT1 expression.

In conclusion, our results provide new insights into the therapy and the management of ITP, confirming ELT also as immune-modulating drug. For the first time, we demonstrated that ELT is able to act as a mediator of macrophage polarization from M1 pro-inflammatory phenotype to M2 anti-inflammatory one, restoring the correct M1/M2 ratio and, consequently, improving the impaired inflammatory profile and immune response in ITP. We demonstrated that ELT induces a reduction of M1 phenotype marker expression levels, decreases pro-inflammatory cytokines release, and inhibits iron intake by reducing DMT1 expression levels, leading to a macrophage switch towards M2 phenotype.

These data strengthen the hypothesis to use ELT not only for the stimulation of platelet production in chronic ITP but also as an immune-modulating drug in patients with newly diagnosed and persistent ITP. In the future, in vitro and in vivo investigations are needed to validate our results and to translate them to the medical practice.

## 4. Materials and Methods

### 4.1. Source of Macrophages

Macrophages were obtained from the peripheral blood of ten ITP children (median age 6 ± 2 years) and ten healthy donors (median age 6 ± 2 years) (Table 1). ITP patients were enrolled in the Department of Women, Child and General and Specialized Surgery of University of Campania Luigi Vanvitelli and in Hematology Department of Bambino Gesù Hospital. They were free from any therapy for at least 15 days. Healthy donors were enrolled in Hematology Department of Bambino Gesù Hospital. 

### 4.2. Macrophages Cultures

Macrophages were obtained from peripheral blood mononuclear cells (PBMCs). PBMCs were isolated by density gradient centrifugation (Ficoll 1.077 g/mL; Lympholyte, Cedarlane Laboratories Ltd., Uden, The Netherlands), diluted at 1 × 10^6^ cells/mL in α-Minimal Essential Medium (α-MEM) (Lonza, Verviers, Belgium) supplemented with 10% fetal bovine serum (FBS) (Euroclone, Siziano, Italy), 100 IU/mL penicillin, and 100 g/mL streptomycin and L-glutamine (Gibco Limited, Uxbridge, UK) and plated in 24-well Cell Culture Multiwell. In order to obtain fully differentiated human macrophages, the PBMCs were cultured for 15 days in presence of 25 ng/mL recombinant human macrophage colony-stimulating factor (rh-MCSF) (Peprotech, London, UK). Culture medium was replaced twice a week. Cells were cultured at 37 °C in a humidified atmosphere with 5% CO_2_. Macrophages derived from ITP patients were treated with ELT (6 µM) from second medium change until the last one. After 15 days of differentiation, cells were harvested for protein extraction, and cell cultures supernatants were collected to perform Iron Assay and to analyze IL-4, IL-10, IL-6, TNF-α, and IFN-γ release with an enzyme-linked immunosorbent assay (ELISA).

### 4.3. Drugs and Treatments

**Figure A1:**
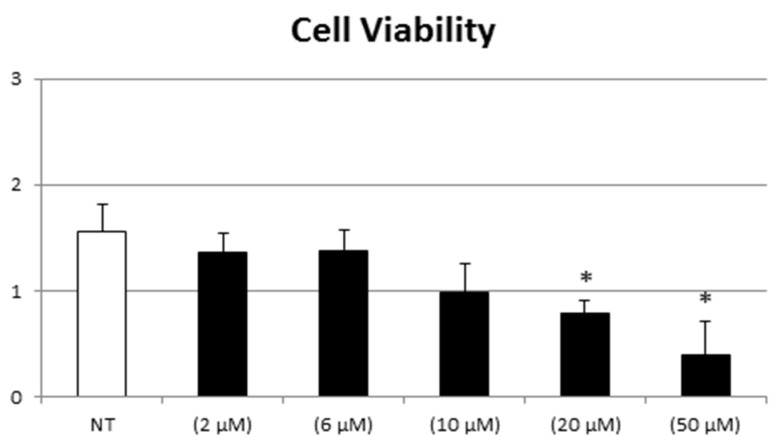
Viability Assay on ITP macrophages treated with different concentration of Eltrombopag. The viability of ITP macrophages was estimated by a cytofluorimetric assay after treatment with Eltrombopag (2, 6, 10, 20, 50 µM). The histogram shows results as cell number × 10^6^ and as mean ± SD of independent experiments on three different patients. * indicates *p* ≤ 0.05 compared to NT.

ELT (Novartis S.p.a., Origgio, VA, Italy) was dissolved in sterile water at a concentration of 10 mM. Cells were treated with ELT at the final concentration of (6 μM). The concentrations of ELT were determined following a pilot Dose-Response experiment (Figure A1). Non-treated cultured cells were maintained in incubation media during the relative treatment time with and without vehicle (sterile water). Lipopolysaccharide (LPS) (Sigma Aldrich, St. Louis, MO, USA) was dissolved in phosphate buffered saline (PBS) containing dimethyl sulfoxide (DMSO). Macrophages obtained from healthy donor were treated with LPS at the final concentration of (500 ng/mL). DMSO final concentration on cultures was 0.01%. Non-treated cultured cells were maintained in incubation media during the relative treatment time with or without vehicle (DMSO 0.01%).

### 4.4. Protein Isolation and Western Blot

Proteins were extracted from treated and non-treated macrophages cultures using radio-immunoprecipitation assay (RIPA) Lysis Buffer (Millipore, Burlington, MA, USA) and following the manufacturer’s instructions. iNOS, CD206, IL-6, and DMT1, proteins were detected in total lysates from cell line cultures by Western Blotting. Membranes were incubated overnight at 4 °C with these antibodies: rabbit polyclonal anti iNOS (1:2000 dilution; Invitrogen, Carlsbad, CA, USA), mouse monoclonal anti CD206 (1:200 dilution; Invitrogen, Carlsbad, CA, USA), rabbit polyclonal anti IL-6 antibody (1:500 dilution; abcam, Cambridge, UK), and mouse monoclonal anti DMT1 antibody (1:100 dilution; Santa Cruz Biotechnology, Dallas, TX, USA). Reactive bands were detected by chemiluminescence (Immobilon Western Chemiluminescent HRP Substrate, Millipore, Burlington, MA, USA) on a C-DiGit^®^ Blot Scanner (LI-COR Biotechnology®, Lincoln, NE, USA). A mouse monoclonal anti β-Tubulin antibody (1:5000, Elabscience, Houston, TX, USA) was used to check for comparable protein loading and as a housekeeping protein. Images were captured, stored, and analyzed using “Image studio Digits ver. 5.0” software.

### 4.5. ELISA

Several ELISA Assay were performed in order to determine IL-6, IL-4, IL-10, TNF-α, and IFN-γ concentration in cell cultures supernatants, by using commercially available Human ELISA Kits (Invitrogen by Thermo Fisher, Waltham, MA, USA) according to the manufacturer’s instructions. Briefly, the microplates were coated with monoclonal antibodies specific to the cytokines. Standards and supernatants were pipetted into the wells of the microplate and were run in duplicate. After the plate was washed, enzyme-linked polyclonal antibodies specific for IL-4, IL-10, IL-6, TNF-α, and IFN-γ were added to the wells. The reaction was revealed by the addition of the substrate solution. The optical density was measured at a wavelength of 450 nm by using the Tecan Infinite M200 (Tecan Group Ltd., Männedorf, Switzerland) spectrophotometer. Cytokines concentrations (pg/mL) were determined against a standard concentration curve.

### 4.6. Iron Assay

After 15 days of differentiation, cell culture supernatants were collected to measure iron (III). The assay was performed by using the Iron Assay Kit (Abcam, Cambridge, UK) according to the manufacturer’s instructions. Briefly, standards and macrophages supernatant were pipetted into the wells and were incubated with an acidic buffer to allow iron release. Then, an iron probe at 25 °C for 60 min was added, protected from light. Released iron reacted with the chromogen resulting in a colorimetric (593 nm) product, proportional to the iron amount. The optical density was measured at a wavelength of 593 nm by using the Tecan Infinite M200 (Tecan Group Ltd., Männedorf, Switzerland) spectrophotometer. Iron (II) and Total Iron (II+III) contents of the test samples (nmol/μL) were determined against a standard concentration curve. Iron (III) content can be calculated as: Iron (III) = Total Iron (II+III) − Iron (II).

### 4.7. Statistical Analysis

Statistical analyses on molecular, biochemical, and cellular data were performed using the Student’s t test (XLSTAT by Addinsoft 2020, Boston, MA, USA) to evaluate differences between quantitative variables. Data are expressed as mean ± SD. A *p* value ≤ 0.05 (* or ^) was considered statistically significant.

## Figures and Tables

**Table 1:** Clinical characteristics of ITP patients.

ITP Patients	
Median age, years (mean ± SD)	6 ± 2
Sex (Female/Male)	6/4
Median Platelet count (mean)	54.3 × 10^9^/L
Platelet count (range)	37–80 × 10^9^/L
Clinical presentation	
Without Fatigue (*n*/10)	10/10
Without bleeding (*n*/10)	6/10
Bleeding limited to skin (*n*/10)	4/10
Without family history of autoimmune disease (*n*/10)	10/10

This table shows the clinical characteristics of 10 enrolled ITP patients in the study.
